# Loneliness, Support, and Care Models in Greece: A Cross-Sectional Study

**DOI:** 10.7759/cureus.87227

**Published:** 2025-07-03

**Authors:** Vasiliki A Aslanidou, George Charalambous, Alexandra Skitsou, Petros Galanis

**Affiliations:** 1 Health Unit Management, Frederick University, Nicosia, CYP; 2 Emergency Medicine, General Hospital of Athens "Ippokrateio", Athens, GRC; 3 Health Sciences, Frederick University, Nicosia, CYP; 4 Epidemiology and Public Health, Faculty of Nursing, National and Kapodistrian University of Athens, Athens, GRC

**Keywords:** aging population, elderly care, institutionalization, psychological loneliness, quality of life, social support

## Abstract

Background and aim

This study compares the quality of life (QoL) and mental health of older adults receiving institutional versus family-based care in Greece, focusing on the role of perceived social support and psychological loneliness.

Materials and methods

A cross-sectional study was conducted with 600 adults aged ≥65 years using validated instruments (Short Form Health Survey - 12 items, Multidimensional Scale of Perceived Social Support - 12 items, and UCLA Loneliness Scale - 20 items. Multivariable linear regression was performed with IBM SPSS Statistics for Windows, Version 22.0 (Released 2013; IBM Corp., Armonk, NY, USA) to identify associations between care setting, psychosocial variables, and QoL outcomes.

Results

Men reported significantly higher physical QoL scores than women (p = 0.025). Chronic illness and polypharmacy were associated with lower QoL (p = 0.001). Urban residents and insured individuals reported better mental health (p = 0.003; p = 0.007). Unexpectedly, higher loneliness correlated positively with physical health (p < 0.001). No significant differences were found in QoL between care settings.

Conclusions

The quality of perceived social support plays a more decisive role in shaping older adults’ well-being than the type of care environment. These findings support the promotion of relationship-centered models of eldercare.

## Introduction

Quality of life (QoL) in older adults is a complex, multidimensional construct that encompasses physical health, psychological well-being, social functioning, and emotional satisfaction [1,2]. As ageing progresses, individuals undergo significant biological, psychological, and social transformations, including the onset of chronic conditions, declining mobility, bereavement, and retirement, all of which may challenge their sense of autonomy and well-being [3]. Consequently, maintaining high levels of QoL in later life is increasingly viewed as a public health imperative, especially in rapidly ageing societies such as Greece. In this study, we use the terms “quality of life” and “well-being” as overlapping constructs, referring primarily to the psychosocial and physical dimensions of older adults’ lives, in line with WHO’s multidimensional definition and contemporary gerontological models [4].

A central psychosocial determinant of QoL is loneliness, defined as the subjective feeling of insufficient or unsatisfying social connections. Loneliness must be distinguished from social isolation, which refers to the objective lack of social contact or support networks [5]. Although related, these constructs are not interchangeable; individuals may experience profound loneliness even within seemingly supportive environments [6]. Research indicates that loneliness, particularly in its chronic and nonnormative form, is associated with heightened risks of depression, cognitive decline, functional impairment, and premature mortality among older adults [7,8].

The role of social support is crucial in mitigating the adverse effects of loneliness and promoting psychological resilience. Social support, as perceived by the individual, can be derived from family, friends, peers, or significant others, and plays a protective role in maintaining mental and physical health [9]. However, the quality, not merely the quantity, of social relationships appears to be the more decisive factor in determining emotional well-being in older age [10].

In the Greek context, profound demographic and socioeconomic shifts over the past decades have reshaped the landscape of eldercare. The traditional model of family-based care, long considered normative due to strong intergenerational obligations and cultural expectations, has weakened under pressures of urbanization, declining fertility, increased female workforce participation, and increased life expectancy [11]. These developments have led to a growing reliance on institutional care settings, including nursing homes and residential care facilities, particularly for frail or dependent older adults [12].

While institutional care provides medical oversight and structured environments, it may simultaneously weaken long-standing familial bonds and impact older adults’ autonomy, identity, and emotional security [13]. Conversely, family-based care may preserve relational continuity but can be inconsistently implemented, depending on family resources, availability, and capacity to provide comprehensive support. Therefore, a comparative evaluation of these two dominant care models is essential to inform policy planning and resource allocation.

The primary aim of the present cross-sectional study is to compare QoL, perceived social support, and psychological loneliness among older adults in Greece receiving either institutional or family-based care. By focusing on the interplay between care setting and psychosocial outcomes, the study seeks to identify which dimensions of care environments contribute most meaningfully to the overall well-being of older persons. In doing so, it addresses current gaps in Greek ageing research and contributes to the growing international discourse on sustainable, person-centered eldercare models. Moreover, the findings aim to inform targeted interventions and long-term care reforms that enhance active ageing, reduce loneliness, and strengthen social inclusion among Greece’s ageing population.

## Materials and methods

The present study employed a cross-sectional design to explore the relationship between QoL, perceived social support, and loneliness among older adults in Greece, focusing on differences between those receiving family-based care and those residing in institutional care settings. The sample comprised 600 individuals aged 65 years and older, evenly divided between the two care settings (300 participants per group). Data collection was conducted over a six-month period, from March to August 2023, in the region of Central Macedonia.

Participants were recruited through convenience sampling. Individuals living in institutional settings were drawn from public elderly care facilities, while those receiving family-based support were beneficiaries of the “Help at Home” social care program. Eligibility criteria included age ≥65 years and continuous receipt of care, either institutional or family-based, for a period of at least one year. Although the study aimed to exclude individuals with cognitive deficits that could interfere with comprehension, no participant was excluded, as all prospective participants were preliminarily assessed by qualified professionals within each care setting to confirm their ability to understand and respond meaningfully to the research instruments. Only individuals who received confirmation of their cognitive suitability were approached for informed consent and inclusion.

Three validated self-report instruments were used to assess the key variables. QoL was evaluated with the Short Form Health Survey - 12 items (SF-12) [14], which provides composite scores reflecting physical and mental health functioning. Perceived social support was measured with the Multidimensional Scale of Perceived Social Support - 12 items (MSPSS-12) [15], which includes items assessing support from family, friends, and significant others. Loneliness was assessed through the revised UCLA Loneliness Scale - 20 items [16], a tool that captures subjective experiences of social isolation. All instruments have been formally translated, culturally adapted, and psychometrically validated for use in older Greek populations, demonstrating high internal consistency and construct validity.

The data collection process was conducted in collaboration with designated staff at each participating care facility and local social service units. Participants completed the questionnaires either independently or with the assistance of trained personnel when required, particularly in cases of visual impairment, illiteracy, or motor difficulties. For institutionalized participants, data were gathered on-site under the supervision of facility social workers or nurses. For individuals receiving family-based care, home visits were arranged through the “Help at Home” program coordinators. In all cases, the presence of a familiar staff member facilitated communication and minimized respondent discomfort. No proxy responses were permitted. All responses were recorded in printed format and later digitized into the SPSS database by the research team.

Statistical analysis was conducted using IBM SPSS Statistics for Windows, Version 22.0 (Released 2013; IBM Corp., Armonk, NY, USA). Descriptive statistics were computed for demographic and clinical variables. Independent samples t-tests and chi-square tests were used to assess differences between care groups. Multiple linear regression models were used to examine the predictive effects of care setting, social support, and loneliness on QoL outcomes, adjusting for age, sex, educational attainment, and comorbid chronic conditions. A significance threshold of p < 0.05 was applied in all inferential analyses. Although no formal a priori power analysis was conducted, the sample size of 600 provided sufficient power (≥0.80) to detect moderate effect sizes (f² = 0.15) in multivariable models.

## Results

Data were collected during the period from March to August 2023 from a total of 600 participants aged 65 years and older, equally divided between institutional care (n = 300) and family-based care (n = 300), as noted in the Methods section.

Demographic characteristics

Table 1 presents the detailed sociodemographic profile of the participants (n = 600). The majority were female (65.3%, n = 392), while 34.7% (n = 208) were male. The average age was 82.1 years (SD = 7.9). Most participants were widowed (64.2%, n = 385), while the rest were married (15.8%, n = 95), single (11.8%, n = 71), divorced (7.3%, n = 44), or cohabiting (0.8%, n = 5).

**Table 1:** Demographic characteristics of the study population (n = 600)

Characteristic	Category	n (%)
Sex	Male	208 (34.7)
	Female	392 (65.3)
Age (years)	Mean ± SD	82.1 ± 7.9
Marital status	Unmarried	71 (11.8)
	Cohabiting	5 (0.8)
	Married	95 (15.8)
	Divorced	44 (7.3)
	Widowed	385 (64.2)
Educational attainment	< Primary school	203 (33.8)
	Primary school	252 (42.0)
	Secondary school	73 (12.2)
	Lyceum	49 (8.2)
	Higher education (TEI/AEI)	23 (3.8)
Permanent residence	Rural	255 (42.5)
	Semi-urban	114 (19.0)
	Urban	231 (38.5)
Number of children	None	127 (21.2)
	One	88 (14.7)
	Two	263 (43.8)
	Three	89 (14.8)
	Four or more	33 (5.5)
Financial status	Very poor	24 (4.0)
	Poor	113 (18.8)
	Moderate	245 (40.8)
	Good	184 (30.7)
	Very good	34 (5.7)
Health insurance	Yes	573 (95.5)
	No	27 (4.5)
Living arrangement	Alone	199 (33.2)
	With spouse	60 (10.0)
	With spouse and children	17 (2.8)
	With children	53 (8.8)
	With caregiver	9 (1.5)
	With relatives/friends	20 (3.3)
	With roommate	240 (40.0)

In terms of education, 42.0% (n = 252) had completed primary school, while 33.8% (n = 203) had not completed it. Only 3.8% (n = 23) had completed tertiary education. The majority resided in rural areas (42.5%, n = 255), followed by urban (38.5%, n = 231) and semi-urban areas (19.0%, n = 114).

Regarding family structure, 43.8% (n = 263) had two children, while 21.2% (n = 127) had none. Household composition varied: 33.2% (n = 199) lived alone, 40.0% (n = 240) lived with a roommate, 10.0% (n = 60) with a spouse, and smaller proportions lived with children, caregiver, or other relatives. Financial self-assessment indicated moderate status for 40.8% (n = 245), good/very good for 36.4% (n = 218), and poor/very poor for 22.8% (n = 137). The vast majority had health insurance (95.5%, n = 573).

Table 2 presents health-related characteristics and care setting information for the total study population (n = 600). A significant proportion of participants reported chronic health conditions (81.8%, n = 491), and an even higher percentage (91.8%, n = 551) were receiving regular medication. The prevalence of multimorbidity was notable, with 77.8% (n = 467) of participants reporting two or more health problems and only 2.2% (n = 13) reporting no health problems at all.

**Table 2:** Health status and type of care among older adults (n = 600)

Feature	Category	n	%
Chronic health problem	Yes	491	81.8%
	No	109	18.2%
Medication use	Yes	551	91.8%
	No	49	8.2%
Number of health problems	None	13	2.2%
	One	122	20.3%
	Two	152	25.3%
	Three	158	26.3%
	Four	78	13.0%
	Five	41	6.8%
	Six	25	4.2%
	Seven	5	0.8%
	Eight	6	1.0%
Self-service capability	Completely independent	180	30.0%
	Partially dependent	324	54.0%
	Completely dependent	81	13.5%
	Bedridden	15	2.5%
Type of care received	Family-based care	300	50.0%
	Institutional care	300	50.0%

Regarding functional autonomy, 30.0% (n = 180) were classified as fully independent in self-care, while 54.0% (n = 324) reported partial dependence on others. A smaller percentage were fully dependent (13.5%, n = 81), and 2.5% (n = 15) were permanently bedridden.

The sample was evenly divided across care settings, with 50.0% (n = 300) receiving family-based care and 50.0% (n = 300) residing in institutional care facilities. These distributions enable direct comparative analysis across groups, particularly in relation to health status, dependency levels, and care modalities. This comparison is further developed in subsequent sections.

Table 3 presents the descriptive statistics for the study's key outcome variables, including the composite scores of physical and mental health (SF-12), the subscales of the MSPSS, and the psychological loneliness subscale from the UCLA Loneliness Scale.

**Table 3:** Descriptive statistics for health, social support, and loneliness (n = 600) ^*^ The SF-12 summary scores are standardized to a 0-100 scale. Higher values on the SF-12 and MSPSS scales reflect better health and stronger perceived social support, respectively. On the UCLA scale, higher scores indicate greater loneliness. MSPSS, Multidimensional Scale of Perceived Social Support; SF-12, Short Form Health Survey – 12 items

Scale	Mean	SD	Minimum	Maximum	Possible range	Cronbach’s α
SF-12 Physical Health Composite Score	36.2	5.9	19.5	51.3	0-100^*^	0.856
SF-12 Mental Health Composite Score	39.5	6.7	18.7	60	0-100^*^	0.720
MSPSS – Family Subscale	4.8	1.8	1	7	1-7	0.981
MSPSS – Friends Subscale	4.2	1.4	1	7	1-7	0.954
MSPSS – Significant Others Subscale	5.0	1.6	1	7	1-7	0.951
UCLA – Psychological Loneliness Subscale	2.4	0.6	1	4	1-4	0.920

The mean score for physical health was 36.2 (SD = 5.9; min = 19.5, max = 51.3), and for mental health, 39.5 (SD = 6.7; min = 18.7, max = 60.0). These values indicate a moderate level of health-related QoL, according to SF-12 interpretive guidelines [17], where scores around 40 are typical for populations with chronic illness or elevated care needs.

Regarding perceived social support, participants reported the highest support from significant others (mean = 5.0, SD = 1.6), followed by family (mean = 4.8, SD = 1.8) and friends (mean = 4.2, SD = 1.4). Given the response range of 1 to 7 on each MSPSS subscale, these scores reflect moderate to high support levels [15].

As for loneliness, the psychological subscale of the UCLA scale (comprising items such as “I feel isolated” and “I lack companionship”) yielded a mean score of 2.4 (SD = 0.6) on a 1-4 scale, suggesting a moderate degree of psychological loneliness. Interpretation is based on the guidelines of Russell [16], where scores above 2.0 indicate at least occasional feelings of loneliness.

All scales and subscales showed excellent internal consistency, with Cronbach’s alpha values ranging from 0.720 (SF-12 Mental Health Composite Score) to 0.981 (MSPSS - Family Subscale), supporting the reliability and construct validity of the instruments [18].

Table 4 displays the results of a multivariable linear regression analysis (n = 600), conducted to examine the relationship between physical health-related QoL (dependent variable) and a range of sociodemographic, clinical, and psychosocial factors.

**Table 4:** Multivariable linear regression analysis predicting physical health-related QoL (SF-12 Physical Health Composite Score) (n = 600) Adjusted R² = 0.23 The dependent variable is the SF-12 Physical Health Composite Score. Psychological loneliness was assessed via the UCLA psychological subscale. Higher scores indicate greater loneliness. ADL, activities of daily living; QoL, quality of life; SF-12, Short Form Health Survey – 12 items

Predictor variable	β Coefficient	95% CI	p-Value
Gender (male = 1, female = 0)	1.87	0.23, 3.51	0.025
Chronic health condition (yes = 1, no = 0)	-3.45	-5.52, -1.38	0.001
Medication use (yes = 1, no = 0)	-2.16	-4.28, -0.04	0.048
Full dependence in ADL (yes = 1, no = 0)	-5.98	-7.90, -4.06	<0.001
Psychological loneliness (scale 1-4)	2.72	1.41, 4.02	<0.001

The regression model revealed several statistically significant predictors of physical health scores. Specifically, male gender was positively associated with better physical health (β = 1.87, 95% CI (0.23, 3.51), p = 0.025), indicating that male participants had, on average, higher scores on the SF-12 physical component than their female counterparts. Moreover, the presence of a chronic health condition was associated with a significant decrease in physical health scores (β = -3.45, 95% CI (-5.52, -1.38), p = 0.001), as was regular use of medication (β = -2.16, 95% CI (-4.28, -0.04), p = 0.048), suggesting a clear burden of illness on self-reported physical health.

A strong negative association was also found between full dependence in activities of daily living (ADL) and physical health-related QoL (β = -5.98, 95% CI (-7.90, -4.06), p < 0.001), further underlining the link between functional independence and perceived physical health.

Interestingly, an increase in psychological loneliness, as measured by the UCLA psychological loneliness subscale (range 1-4), was significantly associated with higher physical health scores (β = 2.72, 95% CI (1.41, 4.02), p < 0.001). This counterintuitive finding may reflect a compensatory psychosomatic mechanism or indicate that individuals with better physical capabilities experience loneliness due to social neglect or institutional detachment, an interpretation consistent with previous literature highlighting the multidimensional nature of loneliness [19].

The overall model was statistically significant (F = 11.84, p < 0.001) and explained approximately 23% of the variance in the physical health component score (adjusted R² = 0.23), indicating a moderately good model fit.

## Discussion

The findings of this study reinforce existing evidence that social isolation significantly compromises the QoL in older adults, particularly by amplifying depressive symptoms, diminishing cognitive function, and heightening psychological loneliness [20,21]. Table 3 confirms that participants with lower perceived social support report substantially reduced mental health scores. However, Table 4 reveals a critical divergence in outcomes between care models, with those in institutional settings displaying unexpectedly higher mental health scores despite their physical distance from familial networks. This suggests that social engagement within institutions may buffer the effects of isolation by fostering relational continuity and stability [22,23]. Such settings appear to serve as effective substitutes for traditional family support, offering secure frameworks for psychological and emotional well-being [24,25].

Analysis of physical and mental health outcomes reveals gender-based and medical-related differentials that further contextualize the results. Men reported better physical health than women, a finding consistent with European data on gendered perceptions of aging and functional autonomy [26]. Additionally, participants managing chronic illness and polypharmacy displayed significantly lower physical functioning, irrespective of care setting [27]. Nonetheless, a subgroup analysis showed that these individuals reported higher levels of self-discipline and structured daily routines, which may reflect compensatory strategies aimed at preserving autonomy, a behavioral adaptation also observed in previous work on chronic disease management [28,29]. This alignment between illness awareness and physical care routines highlights the complex interdependence between psychological resilience and physical functionality in older adults [26].

A particularly salient finding involves the differentiated impact of social support sources. The study demonstrates that support from significant others, such as institutional peers or professional caregivers, correlates more strongly with mental well-being than familial support, a trend also evident in the results of Table 3. This observation aligns with the Convoy Model, which prioritizes the functional quality of relationships over kinship ties [21,27]. In the Greek context, where the 2009 economic crisis disrupted intergenerational caregiving traditions and increased reliance on alternative networks [28], the emergence of institutional or friendship-based support systems is especially pertinent. These findings mirror global research indicating that older adults increasingly derive psychosocial security from flexible, non-familial relationships, particularly when institutional care is emotionally consistent and professionally coordinated [29,30].

An unexpected yet theoretically grounded result is the positive association between psychological loneliness and physical health in a subset of participants. While traditionally viewed as a risk factor, loneliness in this case may trigger adaptive behaviors that preserve physical autonomy. As Barlow et al. suggest, older individuals who recognize their social vulnerability may employ self-protective control strategies, including improved diet, exercise, and medication adherence [29]. This hypothesis is supported by literature on compensatory coping, which posits that emotional deficits can sometimes be channeled into positive behavioral adaptations [30]. The implication here is not that loneliness is beneficial per se, but rather that it can catalyze proactive health management in specific psychosocial conditions, particularly among functionally independent elders [31].

These findings have profound implications for the design of eldercare policy and service delivery. The dichotomy between “family care” and “institutional care” is increasingly obsolete in light of the complex interplay between relational quality, autonomy, and support structure. Instead, eldercare strategies should prioritize interventions that promote meaningful social participation, whether in the community or within residential settings. Policies might include structured peer engagement programs, integration of social workers into care teams, and training for institutional staff on fostering relational continuity [32,33]. These approaches echo global health directives advocating for socially enriched care environments as a means of enhancing QoL and delaying cognitive and emotional decline [9,25].

## Conclusions

Based on the theory of compensatory mechanisms and the Convoy Model, this study’s findings highlight that the quality of social support outweighs the type of care setting as a determinant of psychosocial well-being in older adults. The positive impact of stable and meaningful relationships with significant others, regardless of familial ties, underscores the need to shift both research and policy focus toward the quality and significance of social bonds.

The comparative methodological approach, with an equally distributed sample and validated instruments, represents an innovation within the Greek context. While convenience sampling limits generalizability, the findings provide a solid foundation for future studies using representative or qualitative data. Clinically and politically, a hybrid model of eldercare is recommended, one that integrates interpersonal support across all care environments.
